# The Role of Yoga in Palliative Care: Insights From a Single Case Study

**DOI:** 10.7759/cureus.83993

**Published:** 2025-05-12

**Authors:** Manjunath G N, Ashween Bilagi, Sunitha L, Shriya S, Prabhakar Kamarthy

**Affiliations:** 1 Department of Radiation Oncology, Sri Devaraj Urs Medical College, Kolar, IND; 2 Department of Integrative Medicine, Sri Devaraj Urs Academy of Higher Education and Research, Kolar, IND; 3 Department of General Medicine, Sri Devaraj Urs Medical College, Sri Devaraj Urs Academy of Higher Education and Research, Kolar, IND

**Keywords:** case study, functional assessment of cancer therapy-lung (fact-l) scale, non-invasive, palliative care, yoga and palliative care, yoga therapy

## Abstract

This case study discusses how a month-long, personalized yoga therapy program impacted a 57-year-old woman with lung cancer that had spread to her brain, who is currently receiving palliative care. Her yoga routine included gentle exercises (Sukshma Vyayama), breathing techniques (Pranayama), and meditation, which were suitable to fit her physical abilities. The Functional Assessment of Cancer Therapy-Lung (FACT-L) scale was used to measure her quality of life, revealing significant improvements in her well-being. She showed improvement in mobility, respiratory function, and emotional stability, as well as reduced lung cancer-related symptoms. Yoga's holistic approach not only supported her physical health but also enhanced her emotional and spiritual wellness, leading to a higher quality of life during her care. These results suggest that yoga therapy could be a valuable, non-invasive addition to palliative care, providing comfort and tailored support for patients with terminal illnesses.

## Introduction

Cancer, often referred to as the "silent pandemic," remains one of the leading causes of mortality globally. Beyond the physiological burden, cancer impacts the psychological, emotional, and spiritual dimensions of a patient's life. In this context, palliative care emerges as a crucial component of comprehensive cancer care, aimed at alleviating suffering and enhancing the quality of life through symptom relief, psychological support, and spiritual well-being. Palliative care is particularly vital in advanced stages of cancer and end-of-life situations, where it supports both patients and families through a holistic, patient-centered approach [1].

Complementary therapies such as yoga are increasingly recognized for their potential to support palliative care. Rooted in ancient Indian philosophy, yoga offers practices that address physical discomfort, emotional distress, and spiritual disconnection, dimensions often underserved in conventional medical treatments. Evidence suggests that yoga can enhance quality of life, reduce symptom burden, and promote psychological resilience in cancer patients, particularly in palliative settings. Despite its growing relevance, access to palliative care remains limited. The World Health Organization's 2020 Palliative Care Fact Sheet estimates that about 56.8 million people need palliative care annually, yet only around 14% receive it [2]. In India, where cancer and other chronic illnesses affect millions, only 4% of cancer patients receive adequate palliative care [1]. This gap is more profound in rural areas, where lack of infrastructure, trained professionals, and awareness hampers access to integrative care options such as yoga therapy [3]. Cultural stigma, economic barriers, and fragmented healthcare services further marginalize affected individuals [4-6]. This calls for innovative, culturally rooted, and accessible strategies, such as yoga-based interventions, to bridge this gap in supportive and palliative oncology care.

Over the past decade, oncology has witnessed a paradigm shift toward personalized and patient-centered care, influenced by global and national healthcare reforms, ethical imperatives, and a growing emphasis on quality of life. This transition has been guided by frameworks such as the World Health Organization's People-Centered and Integrated Health Services strategy (2015), which promotes patient empowerment and shared decision-making in all aspects of care [7]. In India, the National Cancer Grid (NCG), established in 2012, has increasingly emphasized standardized, evidence-based, and patient-centric cancer care, incorporating psychosocial and palliative dimensions. Further, the National Health Policy of India (2017) acknowledges the importance of dignity, autonomy, and patient rights, supporting personalized approaches in treatment planning [8]. Research also supports this shift: studies from the early 2010s onward have demonstrated that involving cancer patients in treatment decisions improves treatment adherence, satisfaction, emotional well-being, and overall quality of life for both patients and their caregivers [9]. As a result, shared decision-making and personalized care planning are becoming integral to modern cancer care.

The growing emphasis on personalized care creates an opportunity to integrate holistic approaches such as yoga into palliative care, bridging gaps in fragmented healthcare systems and enhancing overall patient well-being. As a mind-body intervention, yoga acts through multiple physiological and psychological pathways. It modulates the autonomic nervous system by enhancing parasympathetic activity and reducing sympathetic overdrive, thereby improving heart rate variability and reducing stress-related responses [10]. Regular practice of yoga has also been shown to regulate the hypothalamic-pituitary-adrenal (HPA) axis, reducing cortisol levels and inflammatory markers such as interleukin-6 (IL-6) and C-reactive protein (CRP), which are often elevated in cancer patients undergoing distress [11].

Psychologically, yoga fosters emotional resilience by improving mindfulness, body awareness, and coping strategies. Studies indicate that it reduces anxiety, depression, and fatigue while enhancing emotional well-being and spiritual connection, core aspects of quality of life in palliative settings. Integrating such complementary therapies within palliative care not only helps manage symptoms such as pain, fatigue, and insomnia but also promotes a sense of agency and calm, contributing to a more dignified and holistic approach to end-of-life care [12].

Research suggests that incorporating yoga and other mind-body interventions within palliative care frameworks can provide non-pharmacological relief, fostering a sense of empowerment and inner strength, thus making end-of-life care more compassionate for individuals facing terminal illnesses like cancer [13]. Beyond physical improvements, the philosophical aspects of yoga promote acceptance, peace, and dignity in life's final stages, aligning with the core objectives of palliative care [14]. The integration of holistic approaches such as yoga with conventional medical treatments provides critical emotional, social, and spiritual support.

Need for the study

This is the first single case study to specifically address lung cancer with brain metastasis in a rural Indian context, an area that remains underrepresented in existing literature despite bearing a significant disease burden. Rural populations often face compounded challenges such as poor access to palliative care services, limited awareness of complementary therapies, and infrastructural barriers that restrict healthcare delivery [4]. While the integration of yoga into cancer care has been studied in urban and institutional contexts, its application in rural areas remains minimal, where traditional practices like yoga have either faded or are surrounded by cultural stigma. Focusing on rural settings enables the exploration of feasibility, acceptability, and contextual factors that influence the integration of yoga into palliative care. Although this study focuses on a rural case, the insights gained may be extrapolated to inform strategies for other underserved populations facing similar barriers. The study thus aims to bridge gaps between traditional Indian healing systems and modern palliative frameworks, hypothesizing that yoga, as a complementary therapy, can offer substantial therapeutic and psychological benefits when thoughtfully introduced in rural contexts.

Aims and objectives

This study aims to evaluate the impact of an integrated yoga program on the functional well-being and subjective experiences of a patient receiving palliative care. The objectives of this study are to evaluate the impact of integrated yoga on functional well-being in a palliative care patient and to evaluate the impact of integrated yoga on subjective experiences and perceived benefits of yoga in a palliative care patient.

## Case presentation

Mrs. L, a 57-year-old woman from a rural village near Tamaka, Kolar, was diagnosed with carcinoma of the left lung with brain metastasis. At the time of referral to yoga therapy, her chief complaints included persistent fatigue, mild exertional dyspnea, disturbed sleep, intermittent headaches, low mood, reduced appetite, and a general sense of unease. Neurological examination revealed mild left-sided hemiparesis and occasional dizziness, persisting post-surgery. These symptoms adversely impacted her quality of life and daily functioning. Functionally, her Eastern Cooperative Oncology Group (ECOG) Performance Status was 1, indicating she was ambulatory and capable of light work. Her Karnofsky Performance Status (KPS) was 80% post-surgery, denoting the ability to carry on normal activity with effort and some signs of symptoms. She had no prior history of diabetes, hypertension, asthma, or tuberculosis. Her only notable symptom before diagnosis was occasional headaches.

On June 27, 2024, Mrs. L underwent craniotomy with decompression surgery at a tertiary care hospital in Kolar to address symptoms related to brain metastasis. She was admitted for chemotherapy and radiotherapy on July 27, 2024. She received whole-brain radiotherapy (WBRT) delivering a total of 30 Gray (Gy) in 10 fractions, aimed at reducing tumor burden and managing neurological complications. Subsequently, she completed six cycles of palliative chemotherapy comprising injection pemetrexed (500 mg/m²) and injection carboplatin (AUC 5), administered every three weeks starting in August 2024.

Yoga therapy was initiated on August 6, 2024, concurrently with her chemoradiotherapy. It was selected through a structured clinical decision-making process rooted in the Pancha Kosha framework and principles of patient-centered care. A pre-intervention evaluation using a semi-structured intake form and a kosha-based checklist revealed imbalances at the Annamaya (physical) and Pranamaya (vital energy) levels. Bhakti Yoga was prioritized due to its emotional and spiritual relevance to the patient, as well as its suitability for her clinical condition, given its low physical demand. This alignment was established through psychosocial history and a spiritual inclination screening adapted from yoga clinical intake models. The primary goal was to alleviate perceived stress and promote holistic well-being using accessible, individualized yogic tools. Quality of life outcomes were assessed using the Functional Assessment of Cancer Therapy-Lung (FACT-L) scale, a validated instrument widely applied in lung cancer populations [15]. Following the integration of yoga therapy, her KPS improved to 90%, indicating near-normal functioning. She remained independently mobile, engaged in self-care, and resumed light household responsibilities.

Intervention

The intervention protocol consisted of breathing exercises, subtle movements, Pranayama, chanting, and meditation, carefully tailored to the patient's condition. To ensure safety, all standing, balancing, and dynamic yoga practices, including Kapalabhati and Bhastrika Pranayama, were strictly avoided. The patient underwent a structured one-month in-hospital yoga therapy program, conducted directly over her hospital bed. In the initial phase, as the patient was unable to move or sit up, simple supine practices were introduced. As her mobility improved, the full protocol was gradually incorporated. Each session began with breath awareness and concluded with either yogic relaxation or a short meditation session. During the first two weeks, both the patient and her caretaker received guided instruction from a professional yoga therapist. The caretaker, who had some prior familiarity with yoga, was trained to assist the patient post-discharge, ensuring continued practice at home. Following the supervised phase, the patient independently practiced the full yoga module under the investigator's guidance. The protocol was followed twice daily, in the morning and evening, for 40 minutes per session. The complete yoga practice module is detailed in Table 1.

**Table 1:** Yoga therapy protocol

Serial number	Practice name	Rounds	Duration
1	Free observed breath	10	2 minutes
2	Om chanting	8 rounds	2 minutes
Sukshma Vyayama			
1	Greeva Shakti Vikasaka 1, 2, 3, and 4	5+5+5+5	3 minutes
2	Skanda Sanchalana (clockwise and anticlockwise)	5	1 minute
3	Kaphoni Shakti Vikasaka	5	30 seconds
4	Manibandha Shakti Vikasaka	8	30 seconds
5	Anguli Shakti Vikasaka	10	30 seconds
6	Padanguli Shakti Vikasaka	10	30 seconds
7	Gulpha Shakti Vikasaka (forward and backward, lateral bends and rotation of ankles)	5 each	1 minute
8	Janu Shakti Vikasaka	10	30 seconds
10	Kati Shakti Vikasaka (lateral and twisting movements)	5+5	1 minute
Breathing exercises			
1	Hands in and out breathing	5	30 seconds
2	Hands stretch breathing	5+5+5	1 minute
3	Single-leg raise breathing	5+5	1 minute
4	Bridge stretch breathing	5	1 minute
5	Folded leg lumbar stretch breathing	10	1 minute
Pranayama			
1	Nadishuddhi Pranayama	9	3 minutes
2	Bhramari Pranayama	9	3 minutes
Relaxation and meditation			
1	Deep relaxation technique with chanting: A, U, M, AUM		5 minutes
2	Kirtan Kriya meditation		12 minutes
Total duration			40 minutes

Results

After a month of practicing yoga consistently, Mrs. L showed significant improvements in her health and overall well-being, as assessed by the FACT-L scale (Table 2).

**Table 2:** FACT-L results FACT-L: Functional Assessment of Cancer Therapy-Lung

Serial number	Subscale	Pre-scores	Post-scores	% change
1	Physical well-being	0	9	32.14%
2	Social/family well-being	7	20	54.17%
3	Emotional well-being	1	16	62.50%
4	Functional well-being	4	11	25.00%
5	Lung cancer symptoms	8	15	21.88%
FACT-L total		20	71	37.50%

## Discussion

These findings suggest that yoga therapy can significantly enhance the quality of life in palliative care by addressing both physical and emotional well-being. Improvements in Mrs. L's FACT-L scores demonstrated its benefits in alleviating symptoms such as shortness of breath, fatigue, pain, sleep disturbances, and appetite loss, while also enhancing energy levels and mobility. The therapy helped her maintain a degree of independence in daily activities and fostered emotional resilience, enabling her to reconnect with family members and develop a sense of closeness that facilitated healing. Notably, Mrs. L reported a greater sense of acceptance of her condition and reduced fear of death, addressing common emotional challenges associated with terminal illnesses.

Yoga supports cancer patients in palliative care by engaging both physiological and psychological mechanisms. It reduces stress by lowering cortisol levels and activating the parasympathetic nervous system, thereby enhancing relaxation and emotional stability [16]. Improved sleep quality results from melatonin regulation and reduced anxiety through meditative practices [13]. Gentle physical movements in yoga prevent muscle atrophy and joint stiffness, improve respiratory function and oxygenation, and aid in better energy utilization, ultimately reducing fatigue [17]. Additionally, mindfulness practices enhance appetite and digestive function by reducing stress-induced gastrointestinal disruptions. Guided meditation and deep breathing techniques help mitigate panic, restlessness, and existential distress, promoting a calm state of mind in the final stages of life [18].

Previous studies on yoga and cancer care have shown comparable benefits, particularly in breast cancer patients, where yoga improved quality of life, reduced fatigue, alleviated anxiety, and minimized sleep disturbances [19]. As a holistic intervention, yoga addresses the physical, emotional, and spiritual dimensions of care, making it a valuable complement to conventional palliative care [20].

## Conclusions

Yoga therapy provides personalized care by addressing individual health challenges and fostering active patient participation in the healing process. Its adaptability allows modifications to meet changing patient needs, promoting a dynamic approach to palliative care. This flexibility enables patients to achieve a deeper sense of well-being, thereby enhancing their quality of life.

This case study highlights the potential of integrating yoga into cancer care, particularly in rural areas where access to conventional and complementary therapies is limited. Future research should explore the broader application of yoga across diverse populations, including different cancer types and stages, age groups, and sociocultural backgrounds, to assess its generalizability and therapeutic effectiveness. Longitudinal studies are needed to evaluate the sustained impact of yoga interventions on symptom management, emotional resilience, and quality of life throughout the continuum of palliative and end-of-life care. Additionally, comparative effectiveness trials could help determine the optimal types, frequency, and delivery formats (e.g., in-person versus tele-yoga) suited for various clinical settings. Investigating caregiver outcomes, cost-effectiveness, and the role of yoga in interdisciplinary care models would further strengthen the evidence base. Finally, developing context-sensitive ethical guidelines that safeguard patient autonomy, cultural sensitivity, and informed consent will be essential for the responsible integration of yoga into palliative care frameworks.
